# Mutational Analysis and mtDNA Haplogroup Characterization in Three Serbian Cases of Mitochondrial Encephalomyopathies and Literature Review

**DOI:** 10.3390/diagnostics11111969

**Published:** 2021-10-23

**Authors:** Phepy G. A. Dawod, Jasna Jancic, Ana Marjanovic, Marija Brankovic, Milena Jankovic, Janko Samardzic, Ayman Gamil Anwar Dawod, Ivana Novakovic, Fayda I. Abdel Motaleb, Vladimir Radlovic, Vladimir S. Kostic, Dejan Nikolic

**Affiliations:** 1 Faculty of Medicine, University of Belgrade, 11000 Belgrade, Serbia; fibygamilanwer@med.asu.edu.eg (P.G.A.D.); jasna.jancic.npk@gmail.com (J.J.); ana.marjanovic@yahoo.com (A.M.); mara.brankovic@gmail.com (M.B.); ivana.novakovic@med.bg.ac.rs (I.N.); vladimir.radlovic@gmail.com (V.R.); vladimir.s.kostic@gmail.com (V.S.K.); 2 Department of Medical Biochemistry and Molecular Biology, Faculty of Medicine, Ain Shams University, Cairo 11591, Egypt; dr.fayda@hotmail.com; 3 Clinic of Neurology and Psychiatry of Children and Youth, 11000 Belgrade, Serbia; 4 Neurology Clinic, Clinical Center of Serbia, 11000 Belgrade, Serbia; milena.jankovic.82@gmail.com; 5 Institute of Pharmacology, Clinical Pharmacology and Toxicology, Faculty of Medicine, University of Belgrade, 11000 Belgrade, Serbia; jankomedico@yahoo.es; 6 Internal Medicine, Hepatogastroenterology and Endoscopy Department, Faculty of Medicine, Ain Shams University, Cairo 11591, Egypt; ayman.gamil@med.asu.edu.eg; 7 Pediatric Surgery Department, University Children’s Hospital, 11000 Belgrade, Serbia; 8 Physical Medicine and Rehabilitation Department, University Children’s Hospital, Tirsova 10, 11000 Belgrade, Serbia

**Keywords:** MELAS, leigh syndrome, mtDNA, sanger sequencing, mutations, haplogroups

## Abstract

Mitochondrial encephalomyopathies (MEMP) are heterogeneous multisystem disorders frequently associated with mitochondrial DNA (mtDNA) mutations. Clinical presentation varies considerably in age of onset, course, and severity up to death in early childhood. In this study, we performed molecular genetic analysis for mtDNA pathogenic mutation detection in Serbian children, preliminary diagnosed clinically, biochemically and by brain imaging for mitochondrial encephalomyopathies disorders. Sanger sequencing analysis in three Serbian probands revealed two known pathogenic mutations. Two probands had a heteroplasmic point mutation m.3243A>G in the *MT-TL1* gene, which confirmed mitochondrial myopathy, encephalopathy, lactic acidosis, and stroke-like episode syndrome (MELAS), while a single case clinically manifested for Leigh syndrome had an almost homoplasmic (close to 100%) m.8993T>G mutation in the *MT-ATP6* gene. After full mtDNA MITOMASTER analysis and PhyloTree build 17, we report MELAS’ association with haplogroups U and H (U2e and H15 subclades); likewise, the mtDNA-associated Leigh syndrome proband shows a preference for haplogroup H (H34 subclade). Based on clinical–genetic correlation, we suggest that haplogroup H may contribute to the mitochondrial encephalomyopathies’ phenotypic variability of the patients in our study. We conclude that genetic studies for the distinctive mitochondrial encephalomyopathies should be well-considered for realizing clinical severity and possible outcomes.

## 1. Introduction

Mitochondrial encephalomyopathies (MEMP) are clinically and genetically heterogeneous group of neurometabolic disorders resulting from abnormal mitochondrial function [1]. They are represented with various clinical syndromes sharing the oxidative phosphorylation deficiency due to alteration in the enzymes essential to the production of ATP in mitochondria. In general, MEMPs are caused by mutations in genes that control mitochondrial function (mitochondrial or nuclear genome), and can be expressed in many tissues [2]. The unifying feature of MEMP is progressive neurodegeneration of the central nervous system, causing encephalopathy that affects cognition, movement, strength, coordination, sensation, vision, or autonomic control. Non-nervous system tissues such as muscle tissue may also be prominently affected [3,4,5]. These disorders include mitochondrial encephalopathy, lactic acidosis, and stroke like-episodes (MELAS) syndrome, myoclonic epilepsy with ragged red fibers (MERRF) syndrome, Kearns–Sayre syndrome (KSS), maternally inherited Leigh syndrome (MILS), and neuropathy, ataxia, and retinitis pigmentosa (NARP) syndrome. The mtDNA mutations that cause these disorders are generally heteroplasmic, and the age of disease onset is relatively early [6,7,8,9]. More than 200 mtDNA mutations were discovered associated with MEMP and novel mutations are still being reported [10]. Pathogenic mtDNA changes could be maternally inherited or arise de novo [11,12,13].

Leigh syndrome (also called Leigh disease and sub-acute necrotizing encephalomyelopathy, OMIM 25600, LS) is considered the most common progressive and severe neurodegenerative disorder in children with onset within the first months or years of life. LS is characterized by psychomotor regression with progressive loss of mental and movement abilities and may result in fatal encephalopathy [14,15]. The disorder could be associated with mutations in more than 75 genes that have been identified in both the nuclear and mitochondrial genome; about 20% of LS cases are caused by mtDNA mutations [16,17,18]. Point mutations at m.8993T>G or the less severe m.8993T>C in *MT-ATP6* gene in the complex V are the most frequent LS-associated mtDNA changes. LS occurs when mutation load is greater than 90%, with subsequent substitution of the highly evolutionary conserved leucine at position 156 to either an arginine or a proline [19,20,21], affecting the protein component of the F1F0-ATPase that directly blocks ATP generation [22]. Furthermore, both mutations are associated with NARP syndrome when mutation load is around 50–60% [23,24].

MELAS (OMIM# 540000) is an example of MEMPs related to mitochondrial tRNA gene changes. In this syndrome, the predominant mtDNA mutations are in mitochondrial tRNA^Leu^, invariably as heteroplasmics [25,26]. More than 80% of all cases of MELAS are caused by a substitution of m.3243A>G in the *MT-TL1* gene. This nucleotide replacement disrupts the correct 3D folding structure of tRNA^Leu^ by affecting the anticodon wobble base pair of mt-tRNA molecules by reducing the capacity for amino-acylation and methylation. Besides that, this mutation interferes with the 16S RNA molecule transcription and termination site with subsequent accumulation of unprocessed RNA [27,28,29]. Percentages of mutant mtDNA cause different degrees of the energetic defects which are presented clinically by variable phenotypes of MELAS, with central nervous system involvement when the mutant is present at higher percentages including stroke-like episodes, seizures, cortical blindness, and dementia. MELAS is also accompanied by features of myopathy, recurrent headaches, short stature, and episodic vomiting resulting from lactic acidosis [30,31,32].

Several specific mtDNA haplogroups have been associated with different neurodegenerative mitochondriopathies, among them LHON, which is caused by mtDNA mutations [33,34], as well as Parkinson’s disease [35], and Alzheimer’s disease [36], which are associated with mitochondrial dysfunction. Furthermore, haplogroup studies have shown significant roles of both diagnostic features [37] and therapeutic susceptibility [38,39] in various conditions, encouraging targeted mtDNA mutations testing jointly with mtDNA haplogroup determination. However, previous studies did not confirmed the association with MELAS and Leigh syndromes [40].

Here, we present results of the molecular-genetic study for pathogenic mtDNA primary mutations in Serbian probands preliminary diagnosed as MEMP and correlations of genetic data with clinical phenotypes of the probands. Additionally, we analysed mtDNA haplotypes of detected mutations and constructed the phylogenic tree according to previously described concepts [41,42].

## 2. Patients and Methods

### 2.1. Patients

Three Serbian unrelated children included in this study showed features for MEMP syndromes revealed by their clinical evaluation; two of them were suspected for MELAS (proband 1 and proband 2) and one for LS (proband 3). In all cases family history was negative. Probands were recruited from child neurology units at the Clinic for Neurology and Psychiatry for Children and Youth, and Institute for Health Protection of Mother and Child of Serbia, Belgrade, Serbia. Detailed neurological assessments including standardized testing procedures and biochemical and neuroimaging investigations were conducted. All molecular genetic analyses were performed in the laboratory for genetic and molecular diagnostics of neurological disorders, Neurology Clinic, Clinical Centre of Serbia, Belgrade, Serbia.

### 2.2. Ethical Considerations

Conveniently informed consent was obtained from probands’ parents which was then reviewed by the ethical committee (Number: 2650/VI-1, approved on 26 June 2018) of the Faculty of Medicine, University of Belgrade, who provided ethical approval for this study.

### 2.3. Molecular Genetic Methods

DNA for genetic analyses was extracted from 5 mL peripheral blood samples following the manufacturer’s protocol (Invitrogen, Thermo Fisher Scientific, Waltham, MA, USA. Targeted Sanger sequencing to check for m.3243A>G and m.8993T>G pathogenic variants as major causes of MELAS and Leigh syndrome, respectively, was initially performed. PCR was used to amplify specific mitochondrial DNA fragments by using appropriated primers according to Taylor et al., 2001 [43]. MELAS point mutation m.3243A>G is within the mtDNA fragment encompassed with forward primer 5′-GGATCAGGACATCCCGATG-3′ (MT 5F), and a reverse primer 5′-CACCTCTAGCCTAGCCGTT-3′ (MT 5R). Leigh syndrome point mutation m.8993T>G is located inside the mtDNA fragment surrounded with forward primer 5′- ACAATCCTAGGCCTACCCG-3′ (MT 14F), and reverse primer 5′-CCACCAATCACATGCCTATC-3′ (MT 14R). PCR was performed by adding 1 µL of patient DNA sample to a total volume of 12.5 µL solution containing 10× DreamTaq Buffer, 0.2 mM deoxynucleoside triphosphates (dNTPs), 0.5 mM of appropriate both forward and reverse primers, 0.5 U of DreamTaq DNA Polymerase (Thermo Fisher Scientific, Waltham, MA, USA) and 15 µg of Bovine Serum Albumin (BSA). The purified fragments were cleaned up by ExoSAP-enzymatic reaction. Fluorescence-based cycle sequencing was performed by applying a BigDye Terminator v3.1 Cycle Sequencing Kit according to the standard protocols, and more purification was done by alcohol-based nucleic acid ethanol precipitation. Capillary electrophoresis has been used for automated DNA sequencing on ABI Prism 3500 Genetic Analyzer (Applied Biosystems, Waltham, MA, USA). The more detailed description of procedures is previously provided in Dawod et al., 2020 [44].

### 2.4. Haplogroups Analysis and Phylogenetic Tree Reconstruction

Our probands with detected mtDNA primary mutations m.3243A>G and m.8993T>G pathognomonic for LS and MELAS, respectively, underwent entire mtDNA sequencing by using the appreciated primers mentioned in previous studies [44]. We determined the predicted haplogroups for all included probands by MITOMASTER analysis (https://www.mitomap.org/foswiki/bin/view/MITOMASTER/WebHome, accessed on 15 August 2021) [45]. All defined haplotypes were assigned regarding the PhyloTree build 17 for Phylogenetic tree reconstruction (https://www.phylotree.org/ accessed on 25 August 2021) [46].

### 2.5. Bioinformatics Analysis

Alignment and comparison of mtDNA variants were adapted with rCRS “the Revised Cambridge Reference Sequence” (accession NC_012920) by Sequencer DNA Sequence Analysis Software [47]. The MITOMAP database system for the human mitochondrial genome (http://www.mitomap.org/MITOMAP accessed on 15 August 2021) and GenBank for Human Mitochondrial Genome Database (http://www.ncbi.nlm.nih.gov/Genbank/index.html, accessed 15 August 2021) were used for analysis of the detected variants [48,49].

The pathogenicity of nonsynonymous mtDNA sequence changes in mtDNA coding regions have been determined by protein-based metrics using in silico predictive software. We used the Polymorphism PolyPhen-2 database (Polymorphism Phenotyping v2, http://genetics.bwh.harvard.edu/pph2/, accessed on 19 August 2021) and PROVEAN (Protein Variation Effect Analyzer, http://provean.jcvi.org accessed on 19 August 2021) for predicting effects of substitution of amino acids on protein function [50,51]. Meanwhile, PANTHER (Protein ANalysis THrough Evolutionary Relationships, (http://pantherdb.org/panther/summaryStats.jsp accessed on 19 August 2021) was tested as a source for evolutionary history classification of protein sequences [52]. The pathogenic characteristics of mutations in tRNA of mtDNA were evaluated by the MitoTIP scoring system (https://www.mitomap.org/MITOMAP/MitoTipInfo accessed on 19 August 2021) [53]. Furthermore, the Mamit-tRNA database that contains mammalian mitochondrial tRNAs was tested as it provides extensive documentation of polymorphisms and mutations in mitochondrial tRNA genes related to human mitochondrial disorders and deciphering the 2D cloverleaf secondary structures of mitochondrial tRNA (http://mamit-tRNA.u-strasbg.fr accessed on 19 August 2021) [54].

## 3. Results

### 3.1. Mutational Genetic Analysis

**Figure 1:**
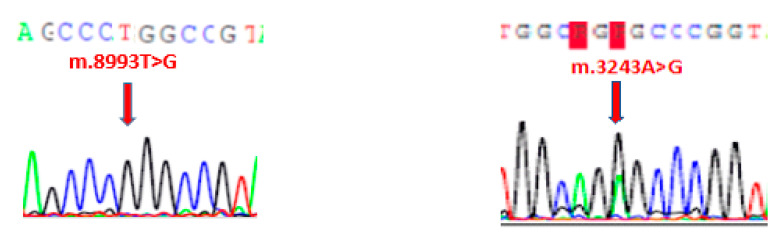
Electropherogram is showing m.8993T>G homoplasmic mutation and m.3243A>G heteroplasmic mutation. Reference sequence is on the top.

Heteroplasmic mtDNA mutation m.3243A>G in the *MT-TL1* gene, which encodes mitochondrial tRNA^Leu^, has been detected in two probands which were clinically corresponding to MELAS diagnosis (proband 1 and proband 2). Mutation m.8993T>G in the *MT-ATP6* gene (F-ATPase protein 6) specific for Leigh disease was detected in proband 3; this mutation was almost homoplasmic (close to 100%) (Figure 1).

In silico predictive software was used for determination of the pathogenic characteristics of mtDNA mutations, nonsynonymous change m.8993T>G in MT-ATP6 gene causing substitution of a hydrophobic leucine residue into a charged arginine residue (L156R) in a highly conserved part of the ATP6 subunit that it has probably damaging effect on the protein function with a score of 0.998 on HumVar Polymorphism PolyPhen-2 database, as well on evolutionary history classification of protein sequences by PANTHER software. Concurrently, PROVEAN showed this amino acid substitution is deleterious on protein function. On another side, different specified informatics predictors were used for assessment of mitochondrial tRNA variant m.3243A>G in the *MT-TL1* gene such as MitoTIP is accessed within MITOMAP, besides, Mamit-tRNA databases, both of them have proven m.3243A>G is a pathogenic mutation in the D-loop of the mt-tRNA^Leu^ with a probably damaging impact on its structure (Table 1).

### 3.2. MITOMASTER Analysis

In our study, entire mtDNA sequencing for all included Serbian probands was carried out for haplogroup analysis, and fasta-formatted files were submitted to MITOMASTER. The results showed that the most frequent MELAS m.3243A>G mutation was associated with both haplogroup H and U, while a single case of mtDNA-associated Leigh syndrome showed predilection for haplogroup H. Furthermore, our analysis reported forty-four polymorphic variants that scattered all over mtDNA fragments at m.55T>C, m.56insC, m.73A>G, m.143G>A, m.152T>C, m.263A>G, m.315insC, m.508A>G, m.739C>T, m.750A>G, m.1438A>G, m.1811A>G, m.2706A>G, m.3116C>T, m.3720A>G, m.3847T>C, m.4769A>G, m.5390A>G, m.5426T>C, m.6045C>T, m.6152T>C, m.6253T>C, m.7028C>T, m.8860A>G, m.10876A>G, m.11197C>T, m.11467A>G, m.11719G>A, m.12308A>G, m.12372G>A, m.13020T>C, m.13359G>A, m.14766C>T, m.15326A>G, m.15519T>C, m.15907A>G, m.15948A>G, m.16051A>G, m.16093T>C, m.16129G>C, m.16183A>C, m.16189T>C, m.16291C>T and m.16519T>C; most of them presented in different frequencies in different haplogroups (Table 2).

### 3.3. Phylogenetic Tree Construction

**Figure 2:**
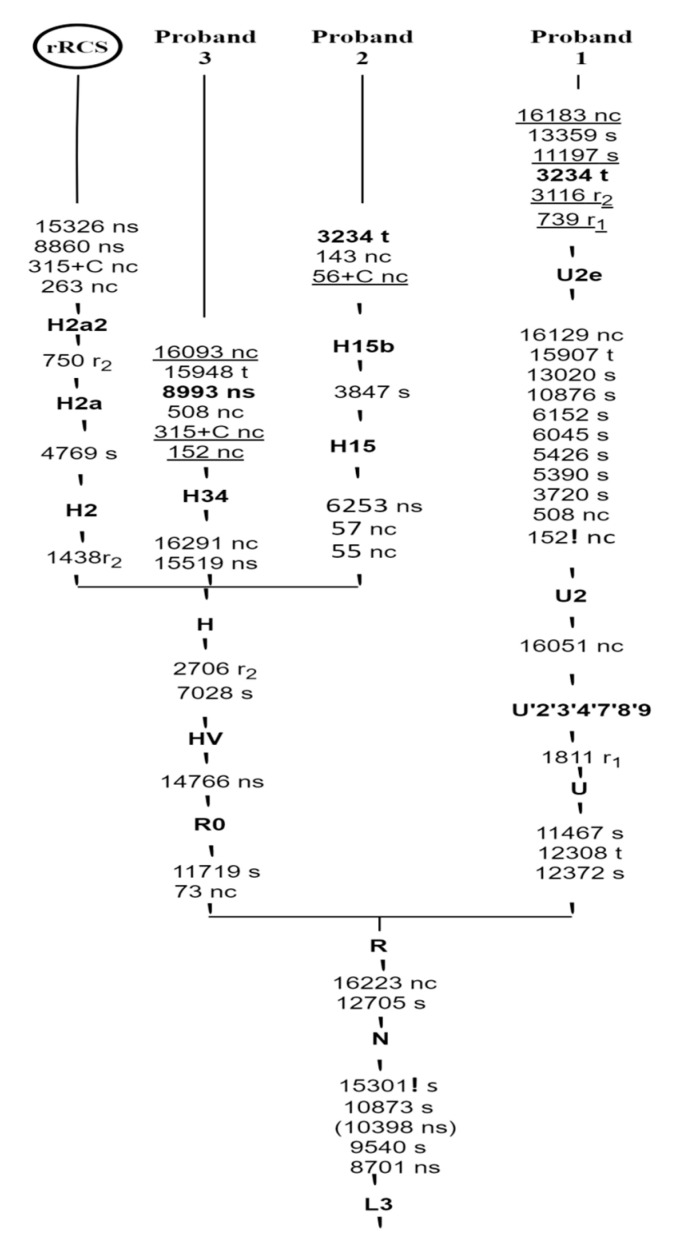
Phylogenetic reconstruction is presenting full mtDNA Sanger sequencing of three Serbian probands have mitochondrial encephalomyopathies mutations. The tree was rooted by following the nomenclature of mtDNA tree Build 17. ! Indicates back mutation, () indicates recurrent mutation; rCRS indicates Revised Cambridge Reference Sequence; nc, s, ns, t, r_1_ and r_2_ indicate non-coding region, synonymous, nonsynonymous, transfer RNA, ribosomal RNA1, and 2 variants, respectively. Primary mitochondrial encephalomyopathies mutations are shown in bold, 3243 for MELAS and 8993 for Leigh syndrome. Underlining indicates variants specific for haplogroups reported on Mitomaster analysis vs. PhyloTree Build 17.

All detected variants are represented for more explication and construction of the phylogenetic tree compared with the rCRS haplogroup (H2a2a) for establishment of the haplogroup affiliation and motifs, following the nomenclature of mtDNA tree Build 17 (Figure 2).

### 3.4. Genotype-Phenotype Relationship

Clinical evaluation, laboratory data and brain imaging of positive m.3243A>G and m.8993T>G probands revealed phenotype features of mitochondrial encephalomyopathies (Table 3). Neurological impairment was the most common feature indicating that two probands (P1 and P2) meet the clinical diagnostic criteria for MELAS with onset during the second decade of life and one single case for Leigh syndrome (P3) with age of onset being within a few months of birth. MELAS probands experienced epileptic seizures, psychosis, muscle weakness, hemiparesis, altered conscious, dementia and associated headache and vomiting, while the Leigh proband presented with symptoms of psychomotor retardation preceded by respiratory viral infection, severe early onset of series epileptic attacks with breaks of 10 min between the attacks up to 30 epileptic attacks per day associated with twitches of the left-sided extremities, sometimes followed by twitches of the left half of the face with deviation of the eyes and the head to the right side, other symptoms of speech delay and muscle weakness.

Neurological assessments were done to estimate frequencies of epileptic attacks, sluggishness of the motor system, muscle weakness, generalized dystonia, dementia, and marked irritability in behaviour. Milestone developmental progression for early childhood assessment was also carried out. Furthermore, laboratory assessment of lactate in blood and cerebrospinal fluid (CSF) was estimated, which showed marked elevation. Brain magnetic resonance imaging (MRI) revealed an expended right ventricle of the brain with an extensive zone of the oedematous cortex and areas of abnormal high signal on Fluid Attenuated Inversion Recovery (FLAIR) images, with changes of T2-weight (T2W) which corresponded to MELAS diagnosis, whereas the specific imaging finding for LS is reported as subcortical necrotizing encephalopathy with symmetrical lesions of basal ganglia, and the brain stem as mesencephalon, tectum, substantia nigra and hypoplasia pons with atrophy of the vermis and cerebellar hemisphere. Necrosis was accompanied by mild lateral ventricular dilatation; moreover, cortex hyperintensity on T2- weighted MR imaging was recorded.

Leigh patients had a severe early onset of epileptic attacks with breaks of 10 min between attacks. Up to 30 epileptic attacks per day had been recorded, which were associated with twitches of the left-sided extremities and sometimes followed by twitches of the left half of the face with deviation of the eyes and the head to the right side. Meanwhile, the MELAS probands experienced psychiatric symptoms, epileptic attacks, muscle weakness and hemiparesis. All probands showed significant laboratory and brain image changes.

## 4. Discussion

Disorders of mitochondrial encephalomyopathies are the most frequent group of inherited neurogenetic disorders, caused by point mutations in mtDNA that disrupt the formation of mitochondrially encoded respiratory chain subunits and therefore cause respiratory chain dysfunction. MEMP mainly presented clinically by different phenotypes [55,56,57]. By direct Sanger sequencing of mtDNA in this study, our genetic analysis reported the first sporadic case of mtDNA-associated Leigh syndrome disclosed in a four year old Serbian girl (P3) who was diagnosed according to the criteria declared by Rahman et al. for a neurodegenerative disease with psychomotor developmental retardation, sluggishness of the motor system, muscle weakness, sever epileptic attacks, nystagmus, dystonia and regression in infancy as a results of basal ganglia and/or brainstem damage accompanied by the characteristic features of hyperintense lesions on T2-weighted on MRI and biochemical lactosidosis [58,59]. By our molecular genetic sequencing, we precisely diagnosed mtDNA-associated Leigh syndrome by our finding of a nearly homoplasmic m.8993T>G mutation that correlates the severity of the disease; m.8993T>G was detected in protein-coding gene *MT-ATP6* causing replacement of the strongly conserved leucine to an arginine at position 156 in complex V with subsequent blocking of the terminal step in oxidative phosphorylation. The pathogenicity of this amino acid substitution in *MT-ATP6* (UniProt ID P00846) was checked according to in silico software predictors; PolyPhen-2 and PANTHER considered m.8993T>G probably damaging, along with PROVEAN L156R prediction, which was deleterious (−5.18). Herein, our case showed irrelevant family history and we could not proceed with complete mtDNA sequence analysis for her family. As it is known, heteroplasmic mutation can be transmitted with different mutation loads between generations, exhibiting inter-individual variation of symptoms in the same family [13,60]. Moreover, phenotypic heterogeneity has been recorded in m.8993T>G carriers [61,62]. Our study supports the literature which displays the occurrence of m.8993T>G mutation in sporadic cases with rapid segregation toward homoplasmy [63,64,65]; it is noticeable in a single generation and reported in about 1/5 LS cases [66]. Other differential diagnoses for LS were excluded [67,68,69].

Furthermore, our results revealed a pathogenic heteroplasmic m.3243A>G mutation in the *MT-TL1* gene with a defect in the protein synthesis of mitochondrial tRNA^Leu^ in two teenage sporadic Serbian children (P1 and P2) who experienced MELAS symptoms. Upon our diagnosis for MELAS cases, it fully fit the Japanese criteria for phenotypic and laboratory findings required for definitive MELAS diagnosis through our reporting of more than two clinical findings of stroke-like episodes, including headache with vomiting, seizures and hemiplegia, which appeared in childhood following a period of normal development with undistinguished family history, plus two evidences of mitochondrial dysfunction detected by high lactate levels in plasma and the decisive molecular finding of m.3243A>G mutation [70]. Otherwise, our MELAS diagnosis did not followed Hirano’s diagnostic criteria by lacking clinical–brain imaging correlation for definite diagnosis of stroke-like episodes [71], which can sometimes be missed within variable ages as mentioned in previous literatures [72,73]; the other systemic symptoms and signs of mitochondrial disorder, such as short stature, diabetes mellitus, deafness, ophthalmoplegia or heart failure were not observed. On the other hand, “silent” m.3243A>G mutation carriers were reported in previous studies; they commonly present with autonomic dysfunction [74] and less neuropsychiatric symptoms [75,76], without an overt full MELAS clinical picture. They have a lower mortality than MELAS probands [77]; for that, strict follow up of their metabolite biomarkers is recommended for predicting their potentiality for MELAS [78] and encouraging a stress-free life and ketogenic diet for keeping healthy mitochondria [79]. In our study, relatives of the probands were unavailable for analysis. The pathogenicity of m.3243A>G in *MT-TL1* was confirmed by software specific for mitochondrial tRNA mutations, MitoTIP recorded m.3243A>G as possibly pathogenic (54.30%), and Mamit-tRNA databases for more detailed tRNA 2-D structures also substantiated m.3243A>G pathogenicity.

The strong genotype–phenotype correlations in MELAS and Leigh disease have been discussed in the literature. Our LS proband’s phenotype is consistent with Sofou et al.’s 2018 study regarding the onset of disease and severity of m.8993T>G mutation in *MT-ATP6* which is preferentially presented with repeated epileptic attacks [80]. Phenotype diversity of Leigh disease has been reported for m.8993T>C mutation in the same *MT-ATP6* gene [81]; noticeably, other unusual presentations rather than neurological ones were found with other mitochondrial and nuclear-encoded genes, causing Leigh disease to be ocular and gastrointestinal with *MT-ND* mutations [82,83], cardiac with *NDUF* [84,85] and renal with *SURF1* and *ACAD9* gene defects [86,87]. Previous studies have reported many mitochondrial mutations causing classic MELAS without any detectable phenotypic specificity [88,89]; exceptional renal diseases were detected in association with m.3243 and m.13513G>A as a first manifestation of MELAS [90,91]. Furthermore, MELAS m.3243 was associated with uncommon presentations such as cardiac and ketoacidosis [92,93].

Evolutionary European mtDNA haplogroups were detected by MITOMASTER analysis in our patients, in which MELAS showed preference for U and H haplogroups with collection of their associated haplotypes. In Proband one, thirty three different homoplasmic sequence variants were identified; twenty eight of them are haplotypes for haplogroup U2e regarding PhyloTree build 17, included defining mutational haplogroup U markers at m.11467A>G, m.12308A>G and m.12372G>A, and ancestral markers motifs at m.73A>G, m.263A>G, m.750A>G, m.1438A>G, m.2706A>G, m.4769A>G, m.7028C>T, m.8860A>G, m.11719G>A, m.14766C>T and m.15326A>G. All were found to be widely distributed across our sample. Furthermore, the characteristic mutation m.1811A>G for U’2’3’4’7’8’9 the common ancestor of haplogroup U was detected, with subdivision to European U2e on the basis of our finding of characteristic non-coding variants at m.152T>C, m.508A>G, m.15907A>G, m.16051A>G, m.16129G>C and m.16189T>C and the synonymous SNPs at m.3720A>G, m.5390A>G, m.5426T>C, m.6045C>T, m.6152T>C, m.10876A>G and m.13020T>C. The other five variants were detected in our analysis at m.739C>T, m.3116C>T, m.11197C>T, m.13359G>A and m.16183A>C, which are considered nonspecific to haplogroup U2e regarding PhyloTree build 17, although all of them with the exception of m.13359G>A have lower frequencies in haplogroup U2e by Mitomaster analysis (0.29%, 22.71%, 22.42% and 75.52%, respectively) (GenBank ID KY930472.1 and AY339545.1); interestingly, three of them, m.739C>T, m.3116C>T and m.13359G>A, were detected as polymorphisms and pathogenic mutations in different other diseases associated with aminoglycoside-induced hearing loss [94,95,96,97], whereas haplotypes for haplogroup H, a subclass of haplogroup HV, were shown to be associated with the second MELAS proband. Ancestral marker motifs were detected at m.263A>G, m.750A>G, m.1438A>G, m.4769A>G, m.8860A>G, m.15326A>G, and m.16519C>T; haplogroup H selected markers were detected at m.2706G>A and m.7028C>T. This MELAS proband was characterized as the H15 subclass, and we detected variants for haplogroup H15 at m.55T>C and m.6253T>C which further subdivided to H15b in the presence of m.3847T>C. In addition we detected two other non-coding variants: insertion C at m.56, which has very low frequency in association to haplogroup H15b on MITOMASTER (GenBank ID KF162889.1), and m.143G>A that did not reported previously in association to that haplogroup. Our results are consistent with European haplogroups [98], and also in agreement with Caucasian and Spanish population studies for MELAS m.3243A>G mutation which reported its association with both the most represented haplogroup H and haplogroup U without any predilection for affecting MELAS phenotypic expression [40,99]; however, another study reported MELAS low representation on haplogroup J in French patients [100]. In contrast to this, the Spanish population did not show any haplogroup preference [40]. Concerning the geographical variations effect, we can notice that the native American haplogroup B2 was exhibited in Mexican MELAS females [101]; likewise, Eastern Asian haplogroups were detected in Chinese MELAS pedigrees [102] and in Indian MELAS patients [103].

The Leigh disease proband exhibited a preference for haplogroup H, subclade H34 corroborated by detection of non-synonymous substitution at m.15519T>C and m.16291C>T. Further, full sequence analysis detected other polymorphic variants m.152T>C, 315insC and 16093T>C not reported on PhyloTree build 17, but published before on Mitomaster haplogroup H34 (GenBank ID JQ702662.1 and KY797254.2). Furthermore, analysis revealed two variants m.508A>G and m.15948 A>G which is not reported on either the PhyloTree build 17 or Mitomaster for that group; the m.15948 in *MT-TT* is a conventional tRNA in the acceptor stem domain of threonine and the transition of A to G is considered possibly benign (29.90%) by MitoTIP. Our results are in line with other previous reported Leigh pedigrees that have showed more preference for subclades of haplogroup H; for example, H1r1 in Spanish pedigree harbouring the LS m.1555A>G Mutation in *MT-RNR1* [104], and an Indian Leigh case study harbouring m.8993T>C mutation in the *MT-ATP6* gene, which was found defining SNP for haplogroup H [105]. Haplogroup heterogeneity for Leigh syndrome has been reported in association with different Leigh-causing mutations; for instance, H13 has been found in association with MILS *ATP6* mutant cell lines. Otherwise, our reporting is not in accordance with other studies that have observed susceptibility of Leigh disease on other haplogroups rather than haplogroup H, such as N9a, B5 and Y in Chinese patients [106,107], and haplogroup U5b on mutant cell lines [108]; all are descended from the macro-haplogroup N. Asian haplogroup M is also reported in another Indian Leigh case as harbouring m.8936T>A in the *MT-ATP6* gene [109].

Herein, our suggestion that haplogroup H may increase risk to Leigh disease is due to the early onset of severe symptoms in our proband according to Hong et al.’s classification, presented with delayed development under 1 year of age, followed by up to 30 epileptic seizure attacks per day and motor weakness [110]; our prospect is also supported by other literatures which indicated that haplogroup H increases the tendency for other neurodegenerative disorders such as Alzheimer’s disease [111,112], Parkinson’s disease [113], Huntington’s disease [114], amyotrophic lateral sclerosis [115] and multiple sclerosis [116], and is also involved in other non-neurological degenerative disorders such as aortic stenosis [117], diabetes mellitus [118] and osteoarthritis [119,120].

## 5. Conclusions

Mitochondrial encephalomyopathies in Serbian children presented with specific phenotypes according to the age of onset should be taken into consideration for molecular genetic screening. Our results underscore the importance of recognizing the pathogenic mtDNA mutations and their related mitochondrial haplogroup background, aiming for better definitive diagnosis and assisting in the development of pathogenicity-based therapeutic approaches.

## Figures and Tables

**Table 1:** Informatics predictors for m.3243A>G and m.8993T>G mutations.

Mitochondriopathies	MELAS	Leigh Disease
Mutation	m.3243A>G	m.8993T>G
Gene	RNA Gene *MT-TL1*	Protein Coding gene *MT-ATP6*
Codon number	−	156
Amino acid change	tRNA ^Leu^	Leu-Arg
Mitomap	Confirmed-Pathogenic	Confirmed-Pathogenic
MitoTIP	Pathogenic	−
Mamit-tRNA	Pathogenic	−
UniProt ID	−	P00846
Polyphen Prediction	−	probably damaging
PANTHER	−	probably damaging
PROVEAN	−	Deleterious (−5.180)

*MT-ATP6*: mitochondrially encoded ATP synthase membrane subunit 6; *MT-TL1*: Mitochondrially Encoded TRNA-Leu (UUA/G) 1; Arg: Arginine; Leu: leucine.

**Table 2:** Polymorphic mtDNA variants detected in Serbian probands with mitochondrial encephalomyopathies.

Probands	Haplogroup	Variants	Locus	Nucleotide Changes	A.A Changes	PhyloTree Build 17 in HG Branch	Mitomaster Frequencies in HG Branch
P1	U2e	73	*MT-CR*	A>G	CR	Reported	96.46%
(MELAS)		152	*MT-CR*	T>C	CR	Reported	90.26%
		263	*MT-CR*	A>G	CR	Reported	96.46%
		508	*MT-CR*	A>G	CR	Reported	94.39%
		739	*MT-RNR1*	C>T	rRNA	Not reported	0.29%
		750	*MT-RNR1*	A>G	rRNA	Reported	100.00%
		1438	*MT-RNR1*	A>G	rRNA	Reported	98.82%
		1811	*MT-RNR2*	A>G	rRNA	Reported	94.39%
		2706	*MT-RNR2*	A>G	rRNA	Reported	99.11%
		3116	*MT-RNR2*	C>T	rRNA	Not reported	22.71%
		3720	*MT-ND1*	A>G	Q138Q	Reported	98.23%
		4769	*MT-ND2*	A>G	M100M	Reported	98.52%
		5390	*MT-ND2*	A>G	M307M	Reported	98.52%
		5426	*MT-ND3*	T>C	H319H	Reported	98.52%
		6045	*MT-COI*	C>T	L48L	Reported	98.52%
		6152	*MT-COI*	T>C	V83V	Reported	98.23%
		7028	*MT-COI*	C>T	A375A	Reported	98.80%
		8860	*MT-ATP6*	A>G	T112A	Reported	99.41%
		10,876	*MT-ND4*	A>G	L39L	Reported	99.11%
		11,197	*MT-ND4*	C>T	G146G	Not reported	22.42%
		11,467	*MT-ND4*	A>G	L236L	Reported	98.82%
		11,719	*MT-ND4*	G>A	G320G	Reported	99.40%
		12,308	*MT-TL2*	A>G	tRNA	Reported	98.80%
		12,372	*MT-ND5*	G>A	L12L	Reported	99.70%
		13,020	*MT-ND5*	T>C	G228G	Reported	99.11%
		13,359	*MT-ND5*	G>A	M341M	Not reported	0.00%
		14,766	*MT-CYB*	C>T	T7I	Reported	99.70%
		15,326	*MT-CYB*	A>G	T194A	Reported	99.70%
		15,907	*MT-TT*	A>G	tRNA	Reported	98.82%
		16,051	*MT-CR*	A>G	CR	Reported	96.75%
		16,129	*MT-CR*	G>C	CR	Reported	95.28%
		16,183	*MT-CR*	A>C	CR	Not considered	75.52%
		16,189	*MT-CR*	T>C	CR	Reported	84.36%
P2	H15	55	*MT-CR*	T>C	CR	Reported	63.33%
(MELAS)		56	*MT-CR*	insC	CR	Not reported	6.67%
		143	*MT-CR*	G>A	CR	Not reported	0.00%
		263	*MT-RNR1*	A>G	CR	Reported	80.00%
		750	*MT-RNR1*	A>G	rRNA	Reported	100.00%
		1438	*MT-RNR2*	A>G	rRNA	Reported	96.70%
		2706	*MT-ND1*	A>G	rRNA	Reported	95.60%
		3847	*MT-ND2*	T>C	L181L	Reported	96.67%
		4769	*MT-COI*	A>G	M100M	Reported	96.70%
		6253	*MT-COI*	T>C	M117T	Reported	96.67%
		7028	*MT-ATP6*	C>T	A375A	Reported	97.80%
		8860	*MT-CYB*	A>G	T112A	Reported	96.67%
		15,326	*MT-CR*	A>G	T194A	Reported	96.70%
P3	H34	152	*MT-CR*	T>C	CR	Not reported	90.91%
(LS)		263	*MT-CR*	A>G	CR	Reported	100.00%
		315	*MT-CR*	insC	CR	Not reported	45.46%
		508	*MT-RNR1*	A>G	CR	Not reported	0.00%
		750	*MT-RNR1*	A>G	rRNA	Reported	100.00%
		1438	*MT-RNR1*	A>G	rRNA	Reported	100.00%
		4769	*MT-ND2*	A>G	M100M	Reported	100.00%
		8860	*MT-ATP6*	A>G	T112A	Reported	100.00%
		15,326	*MT-CYB*	A>G	T194A	Reported	100.00%
		15,519	*MT-CYB*	T>C	L258P	Reported	100.00%
		15,948	*MT-TT*	A>G	tRNA	Not reported	0.00%
		16,093	*MT-CR*	T>C	CR	Not reported	45.46%
		16,291	*MT-CR*	C>T	CR	Reported	90.91%
		16,519	*MT-CR*	T>C	CR	Reported	100.00%

This table shows haplogrouping analysis for two MELAS probands (P1 and P2) and one Leigh syndrome proband (P3). Mitomaster analysis for complete mtDNA sequences detected numerous mtDNA haplotypes for haplogroups (U2e, H15 and H34) in different frequencies, of which almost all are reported on the PhyloTree build 17 in specific haplogroup branches, with the exception of m.16183A>C, which was not considered for phylogenetic reconstruction. A.A: amino acid; HG: haplogroup; LS: Leigh syndrome; MELAS: mitochondrial myopathy, encephalopathy, lactic acidosis, and stroke-like episodes; *MT-ATP6*: mitochondrially encoded ATP synthase membrane subunit 6; *MT-CO1*: mitochondrially encoded cytochrome c oxidase 1; *MT-CR:* mitochondrial control region; *MT-CYB*: mitochondrially encoded cytochrome b; *MT-ND1*, *MT-ND2*, *MT-ND3*, *MT-ND4* and *MT-ND5*: mitochondrially encoded NADH:Ubiquinone Oxidoreductase Core Subunit 1, 2, 3, 4 and 5 respectively; *MT-RNR1* and *MT-RNR2*: Mitochondrially Encoded 12S and 16S, respectively; *MT-TL2*: mitochondrially encoded tRNA leucine; *MT-TT*: mitochondrially encoded tRNA threonine; P: proband.

**Table 3:** Clinical evaluation for MELAS and Leigh probands.

Clinical Evaluation	MELAS		Leigh Disease
Proband	P1	P2	P3
Gender	Male	Male	Female
Age at onset of the disease	14 years old	12 years old	Few months after birth
Duration of the disease	1 month	9 years	4 years
Family history of MEMP	−	–	–
Epileptic seizures	+	+	+
Psychosis	+	+	−
Psychomotor retardation	–	−	+
Confusion	+	−	−
Behaviour changes	+	+	+
Dementia	−	+	−
Episodes like stroke	−	−	−
Headache	+	−	−
Eye deviation during seizures	−	−	+
Speech delay	−	−	+
Hemiparesis	+	+	−
Muscle weakness	+	+	+
Muscle twitches	+	−	+
Associated vomiting	+	+	−
Preceding infection	−	+	+
Lactate acidosis	+	+	+
MRI changes	+	+	+

## Data Availability

Not applicable.
